# Use of Farmers Markets by Mothers of WIC Recipients, Miami-Dade County, Florida, 2011

**DOI:** 10.5888/pcd10.120178

**Published:** 2013-06-13

**Authors:** Benjamin M. Grin, Tamara L. Gayle, Diana C. Saravia, Lee M. Sanders

**Affiliations:** Author Affiliations: Benjamin M. Grin, Tamara L. Gayle, Diana C. Saravia, University of Miami Miller School of Medicine, Miami, Florida.

## Abstract

**Introduction:**

Farmers market-based interventions, including the Farmers’ Market Nutrition Program of the Special Supplemental Nutrition Program for Women, Infants, and Children (WIC), represent a promising strategy for improving dietary behaviors in low-income communities. Little is known, however, about the health-related characteristics of low-income parents who frequent farmers markets in urban settings. The objective of this study was to examine the relationship between family-health factors and the use of farmers markets by mothers of WIC recipients.

**Methods:**

We recruited a convenience sample of mothers of children seeking care at a primary care clinic in a large urban public hospital in Miami, Florida, in 2011 (n = 181 total). The clinic was adjacent to a newly established farmers market at the hospital. Each mother completed an interviewer-administered survey that included self-reported measures of maternal and child health, acculturation, dietary behaviors, food insecurity, and use of farmers markets.

**Results:**

Reported use of farmers markets was independently associated with maternal history of diabetes (odds ratio [OR], 6.9; 95% confidence interval [CI], 1.3–38.3) and increased maternal vegetable (but not fruit) consumption (OR, 3.5; 95% CI, 1.5–8.1). Intended future use of farmers markets was independently associated with being unemployed (OR, 2.4; 95% CI, 1.0–5.7), increased maternal vegetable consumption (OR, 2.5; 95% CI, 1.1–5.7), and food insecurity (OR, 3.6; 95% CI, 1.3–10.3).

**Conclusions:**

This study provides a snapshot of factors associated with farmers market use in a diverse population of urban low-income families. Understanding these factors may inform public health approaches to increase fresh fruit and vegetable consumption in communities at high risk for preventable chronic conditions.

## Introduction

Though the health benefits of fruit and vegetable consumption are well documented (1), there remain significant disparities in fruit and vegetable consumption among low-income and racial/ethnic minority populations. In 2000, only 19.8% of US adults without a high school degree reported consuming fruits and vegetables 5 or more times per day, compared with 29.8% of US college graduates (2). Low-income households face many barriers to accessing healthy foods, including cost and neighborhood environmental factors (3,4) An exception to this pattern is the so-called “Latino immigrant paradox,” in which first-generation immigrants often have better health behaviors and diet-related health outcomes than their income-matched peers from more acculturated populations (5).

Previous community-based research has found successful strategies to increase access to healthful food options for low-income populations, and neighborhood farmers markets may present a valuable resource. Congress established the Farmers’ Market Nutrition Program (FMNP) in 1992 to provide mothers in the Special Supplemental Nutrition Program for Women, Infants, and Children (WIC) with coupons for purchasing fresh fruits and vegetables at local farmers markets (6).

Prior studies looking at farmers market use in low-income populations have focused on barriers and incentives to using this resource, identifying factors like cost and location of farmers markets as barriers (7,8). Analysis of sociodemographic characteristics of farmers market users has been limited and primarily used data from random consumer telephone surveys (9,10).

This study was developed in the context of a new community-based innovation: a farmers market co-located with a community health and WIC center. Farmers market–based interventions represent a promising strategy for improving dietary behaviors in low-income communities, particularly among families at high risk for diet-related health conditions including diabetes and cardiovascular disease. Yet little is known about the health-related characteristics of low-income parents who frequent farmers markets in urban settings. The objective of this study was to examine the relationship between family-health factors (including demographics, food insecurity, and maternal health status) and the use of farmers markets by mothers of WIC recipients.

## Methods

This cross-sectional study enrolled a convenience sample of 181 mothers of children aged 5 years or less presenting for care at a primary-care pediatric clinic colocated in a public hospital adjacent to a newly established farmers market in Miami, Florida. At the time of the study, regional growers were engaged in a collaborative effort with the co-located WIC program through the US Department of Agriculture to facilitate use of WIC vouchers for the purchase of fresh fruits and vegetables from market vendors. The study was conducted between October 2010 and June 2011. The study protocol was approved by the University of Miami Human Subjects Research Office (institutional review board protocol no. 20100447).

To be eligible for inclusion in the study, potential participants were required to be aged 18 years or older, English- or Spanish-speaking, and current or former participants in the WIC program. Members of the research team recruited participants from the pediatric primary-care clinic waiting room.

The primary care clinic is in a large public hospital that serves as the major safety net provider of health care services in Miami-Dade County. Miami-Dade residents face substantial environmental stressors, including high adult and child poverty rates (11) and low rates of being insured (12). The pediatric clinic from which participants were recruited serves a diverse population of low-income people, including racial/ethnic minority populations and many Spanish speakers.

At the times of recruitment, study team members approached each woman in the waiting room who was aged 18 or older, explained the purpose of the study, and screened for eligibility. All participants who completed the survey received $10 Target gift cards as compensation for their time. Reported reasons for nonparticipation included lack of time, lack of interest in the study, nonfluency in English or Spanish, and never having been enrolled in the WIC program. We did not collect complete data on participation rates among mothers.

After granting informed consent, research team members asked study participants to complete a 75-item, interviewer-administered survey in either English or Spanish. The interview took between 30 and 45 minutes to complete. We adapted most survey items from previously validated survey instruments covering the following domains: sociodemographic characteristics (13), acculturation (14), English language proficiency (14), health problems (15), health status (15), weight status (15), dietary practices (16), health behaviors (16), food insecurity (17), and health beliefs (18). We developed additional items in an iterative process that included pretesting with the study population, including measures of participant’s use and intended use of farmers markets.

The primary outcome variable was maternal use of farmers markets. We asked participants (yes/no), “A farmer’s market is a market where farmers sell fruits and vegetables directly to the public. Have you visited any farmer’s markets in the past year?” A secondary outcome variable was maternal intention to use farmers markets. We asked participants, “How likely are you to visit a farmer’s market in the next month?” We created a binary variable: those who responded “extremely likely” or “very likely” were considered likely to use farmers markets, and those who responded “somewhat likely” or “not likely” were considered unlikely to use farmers markets.

We considered inclusion of other outcome measures, including use or redemption of WIC farmers market vouchers by participants, but did not include these items in the final survey version. Pretesting of alternative outcome measures with the study population indicated that although many WIC mothers shopped at local farmers markets, no mothers we talked to had ever heard of or used WIC FMNP vouchers. At the time of the study, many local farmers markets in Miami-Dade County did not yet accept WIC FMNP vouchers, and the WIC FMNP program at the co-located hospital farmers market was still in the early pilot phase. Given this community context, we decided to focus our study on the association between broader family-health factors and farmers market participation in this low-income community rather than on the efficacy of particular intervention strategies.

The final analytic sample included 174 out of the 181 mothers recruited into the study. We excluded 7 surveys because the respondent was later found to be ineligible or because of missing data for the primary or secondary outcome variables.

We used the Statistical Package for the Social Sciences version 19.0 (IBM SPSS Statistics for Windows, IBM Corp, Armonk, New York) for all analyses. We compared variables measuring maternal health, acculturation, diet, physical activity, and demographic characteristics by self-reported use of farmers markets and intention to use farmers markets using univariate logistic regression. All variables with a significant bivariate association (*P* value < .20), along with variables theorized to have clinical importance, were considered for inclusion in multivariate logistic regression models for the 2 outcomes: maternal use of farmers markets and maternal intention to use farmers markets. When we found 2 variables to be highly correlated, we included only 1 in the final regression models. We included the following variables in the final regression models: maternal race, marital status, maternal place of birth, maternal education level, maternal employment status, access to transportation, maternal diabetes, maternal high cholesterol, maternal body mass index, maternal perceived health status, maternal vegetable consumption, and 2 measures of family food security.

## Results

Mean age of participants was 29.0 years (SD 8.0) (Table 1). The median age of their children was 12.0 months (range, 0.3–372.0). The most common self-reported racial/ethnic identity of respondents was Hispanic (35.1%) and black (63.2%). About half of respondents were born in the United States. A substantial proportion (20.7%) had not completed high school. About 3 in 4 were enrolled in the WIC program. Among participants reporting no current WIC enrollment, all reported previous WIC enrollment when their child or children were younger. Thirty-five percent reported having visited a farmers market in the last year, and 30.5% said they were likely to visit a farmers market in the next month. Sixty-six percent of respondents were overweight or obese, based on self-reported maternal height and weight. About 1 in 4 mothers reported eating vegetables 2 or more times per day. A substantial proportion of study participants were food insecure. About half reported being concerned about running out of food for their families at some point in the past month.

**Table 1:** Sociodemographic and Family Factors in Study Sample (N = 174), Miami-Dade County, Florida, 2011

Variables	Sample^a^
**Sociodemographic characteristics**	
Age of mother, y, mean (SD)	29.0 (8.0)
Age of youngest child in months, median (range)	12.0 (0.3–372.0)
**Marital status**	
All others	53 (30.5)
Single	121 (69.5)
**Race^b^**	
Black	110 (63.2)
Nonblack	60 (34.5)
**Ethnicity**	
Hispanic	61 (35.1)
Non-Hispanic	113 (64.9)
**US-born**	
Yes	90 (51.7)
No	84 (48.3)
**Education**	
Less than high school graduate	36 (20.7)
High school graduate or higher	138 (79.3)
**Employment status**	
Unemployed	107 (61.9)
Employed	66 (38.2)
**Current WIC enrollee^c^**	
Yes	137 (78.7)
No	37 (21.3)
**Transportation to clinic^d^**	
Bus and all others	100 (57.4)
Car	70 (40.2)
**Number of children in home^e^**	
0–2 children	108 (62.1)
≥3 children	64 (36.8)
**Farmers market participation**	
Visited farmers market in past year	61 (35.1)
Likely to visit farmers market in next month	53 (30.5)
**Maternal health status**	
**Hypertension**	32 (18.4)
**Diabetes**	10 (5.8)
**High cholesterol**	10 (5.8)
**Stroke**	3 (1.7)
**Heart disease**	3 (1.7)
**Overweight or obese (BMI^f^ >25.0)**	114 (65.5)
**BMI, mean (SD)**	29.4 (8.4)
**Currently pregnant**	15 (8.6)
**Currently breastfeeding**	37 (21.8)
**Self-described health**	
Excellent or very good	68 (39.1)
Good, fair, or poor	106 (60.9)
**Maternal health behaviors**	
**Maternal vegetable consumption**	
Eat vegetables ≥2 times per day in the past week	44 (25.3)
Eat vegetables <2 times per day in the past week	130 (74.7)
**Maternal fruit consumption**	
Eat fruits ≥2 times per day in the past week	52 (29.9)
Eat fruits <2 times per day in the past week	122 (70.1)
**Family food security**	
**“It is my job as a parent to keep healthy food for my child”**	
Strongly agree	156 (89.7)
All others	18 (10.3)
**“Feeding my child healthy food is sometimes too expensive”**	
Strongly agree	25 (14.4)
All others	149 (85.6)
**“I have difficulty getting to a store where I can buy fresh fruits or vegetables for my child”**	
Strongly disagree	99 (56.9)
All others	75 (43.1)
**“We were worried whether our food would run out”^g^**	
Often or sometimes true	90 (51.7)
Never true	82 (47.1)
**“The food that we bought just didn’t last”^h^**	
Often or sometimes true	78 (44.8)
Never true	95 (54.6)
**“We couldn't afford to eat balanced meals”^i^**	
Often or sometimes true	67 (38.5)
Never true	105 (60.3)

Abbreviations: SD, standard deviation; BMI, body mass index; WIC, Special Supplemental Nutrition Program for Women, Infants and Children.

a Values are presented as n (%) unless otherwise indicated.

b Percentages do not sum to 100% because those who responded don’t know/not sure or refused response to this item (n = 4) were excluded from analysis.

c All respondents who were not currently WIC enrollees reported prior enrollment in the WIC program.

d Percentages do not sum to 100% because those who responded don’t know/not sure or refused response to this item (n = 4) were excluded from analysis.

e Percentages do not sum to 100% because those who responded don’t know/not sure or refused response to this item (n = 2) were excluded from analysis.

f Calculated from self-reported height and weight, kg/m^2^.

g Percentages do not sum to 100% because those who responded don’t know/not sure or refused response to this item (n = 2) were excluded from analysis.

h Percentages do not sum to 100% because those who responded don’t know/not sure or refused response to this item (n = 1) were excluded from analysis.

i Percentages do not sum to 100% because those who responded don’t know/not sure or refused response to this item (n = 2) were excluded from analysis.

After adjusting for demographic variables and potential confounders, mothers with a history of type 2 diabetes mellitus were 6.9 times as likely (95% confidence interval [CI], 1.3–38.3) to report having visited a farmers market in the past year as mothers without diabetes (Table 2). Increased vegetable consumption was also independently associated with farmers market participation; high vegetable consumers (>2 servings per day) were 3.5 times as likely (95% CI, 1.5–8.1) to visit farmers markets as mothers who consumed fewer vegetables. Bivariate analysis showed that foreign-born mothers were more likely to visit farmers markets than US-born mothers (OR, 2.0; 95% CI, 1.0–3.7), but this relationship was not significant in the multivariate model.

**Table 2:** Bivariate and Multivariate Correlates of Use of Farmers Market (n = 162), Miami-Dade County, Florida, 2011

Variable	Unadjusted Odds Ratio (95% CI)	Adjusted Odds Ratio (95% CI)	*P* Value^a^
**Sociodemographic characteristics**			
**Marital status**			
All others	1.87 (0.97–3.65)	1.78 (0.73–4.33)	0.20
Single	1 [Reference]		
**Race**			
Black	1 [Reference]		
Nonblack	1.99 (1.04–3.84)	1.91 (0.81–4.51)	0.14
**US-born**			
Yes	1 [Reference]		
No	1.95 (1.04–3.67)	1.17 (0.45–3.06)	0.74
**Education**			
Less than high school graduate	1.91 (0.91–4.03)	1.52 (0.60–3.82)	0.38
High school graduate or higher	1 [Reference]		
**Employment status**			
Unemployed	1.54 (0.79–2.97)	1.21 (0.54–2.75)	0.63
Employed	1 [Reference]		
**Transportation to clinic**			
Bus and all others	1.79 (0.92–3.47)	1.67 (0.75–3.69)	0.21
Car	1 [Reference]		
**Maternal health status**			
**Diabetes**			
Diabetes	8.4 (1.7–40.8)	6.93 (1.25–38.37)	0.03
No diabetes	1 [Reference]		
**Cholesterol**			
High cholesterol	3.0 (0.81–11.1)	1.27 (0.26–6.29)	0.77
No high cholesterol	1 [Reference]		
**Weight**			
Overweight or obese (based on BMI calculated from self-reported height and weight)	1.79 (0.90–3.56)	1.39 (0.59–3.06)	0.47
Not overweight or obese	1 [Reference]		
**Self-described health**			
Excellent or very good	1 [Reference]		
Good, fair, or poor	1.36 (0.71–2.59)	1.20 (0.54–2.69)	0.66
**Maternal health behaviors**			
Eat vegetables ≥2 times per day in the past week	2.65 (1.31–5.35)	3.46 (1.48–8.11)	0.004
Eat vegetables <2 times per day in the past week	1 [Reference]		
**Family food security**			
**“Feeding my child healthy food is sometimes too expensive”**			
Strongly agree	2.28 (0.97–5.37)	1.83 (0.62–5.37)	0.27
All others	1 [Reference]		
**“We were worried whether our food would run out”**			
Often or sometimes true	1.61 (0.85–3.05)	1.90 (0.87–4.15)	0.11
Never true	1 [Reference]		

Abbreviations: CI, confidence interval; BMI, body mass index.

a By multiple-variable logistic regression, with “reported use of farmer’s market” as the dependent variable and the following as independent variables: maternal race, marital status, maternal place of birth, maternal education level, maternal employment status, access to transportation, maternal diabetes, maternal high cholesterol, maternal body mass index, maternal perceived health status, maternal vegetable consumption, and food insecurity.

After adjustment for other variables, mothers with an intention to visit farmers markets were significantly more likely than those who did not intend to visit to report food insecurity, namely that “feeding my child healthy food is sometimes too expensive” (OR, 3.6; 95% CI, 1.3–10.3) (Table 3). Maternal unemployment was also independently associated with an intention to visit farmers markets. Unemployed mothers were 2.4 times as likely (95% CI 1.0–5.7) to express an intention to visit farmers markets when compared with employed mothers. Increased vegetable consumption was independently associated with an intention to visit farmers markets; high-vegetable consumers were 2.5 times more likely (95% CI, 1.1–5.7) to express an intention to go to farmers markets compared with those who consumed fewer vegetables. Bivariate analysis showed that mothers without access to a car for transportation were significantly more likely to express an intention to go to farmers markets (OR 2.5; 95% CI, 1.2–5.0); however, this association was not significant in the multivariate model.

**Table 3:** Bivariate and Multivariate Correlates of Intention to Use Farmers Markets (n = 162), Miami-Dade County, Florida, 2011

Variable	Unadjusted Odds Ratio (95% CI)	Adjusted Odds Ratio (95% CI)	*P* Value^a^
**Sociodemographic characteristics**			
**Marital status**			
All others	0.65 (0.31–1.37)	0.66 (0.25–1.70)	0.39
Single	1 [Reference]		
**Race**			
Black	1 [Reference]		
Nonback	1.12 (0.57–2.23)	1.22 (0.50–2.95)	0.66
**US-born**			
Yes	1 [Reference]		
No	1.17 (0.45–1.64)	0.91 (0.34–2.42)	0.86
**Education**			
Less than high school graduate	1.18 (0.54–2.59)	0.84 (0.33–2.12)	0.71
High school graduate or higher	1 [Reference]		
**Employment status**			
Unemployed	2.34 (1.13–4.82)	2.42 (1.02–5.73)	0.04
Employed	1 [Reference]		
**Transportation to clinic**			
Bus and all others	2.45 (1.20–4.99)	1.96 (0.88–4.39)	0.10
Car	1 [Reference]		
**Maternal health status**			
**Diabetes**			
Diabetes	2.41 (0.67–8.73)	2.76 (0.61–12.48)	0.19
No diabetes	1 [Reference]		
**Cholesterol**			
High cholesterol	1.53 (0.42–5.69)	1.11 (0.20–6.21)	0.90
No high cholesterol	1 [Reference]		
**Weight**			
Overweight or obese (based on BMI calculated from self-reported height and weight)	1.17 (0.59–2.32)	1.36 (0.60–3.06)	0.46
Not overweight or obese	1 [Reference]		
**Self-described health**			
Excellent or very good	1 [Reference]		
Good, fair, or poor	0.77 (0.40–1.49)	0.62 (0.28–1.40)	0.25
**Maternal health behaviors**			
Eat vegetables ≥2 times per day in the past week	2.45 (1.20–5.00)	2.46 (1.07–5.66)	0.04
Eat vegetables <2 times per day in the past week	1 [Reference]		
**Family food security**			
**“Feeding my child healthy food is sometimes too expensive”**			
Strongly agree	2.95 (1.24–7.00)	3.62 (1.27–10.33)	0.02
All others	1 [Reference]		
**“We were worried whether our food would run out”**			
Often or sometimes true	1.36 (0.71–2.63)	1.27 (0.59–2.75)	0.61
Never true	1 [Reference]		

Abbreviations: CI, confidence interval. BMI, body mass index.

a By multiple-variable logistic regression, with “intention to use farmer’s market” as the dependent variable and the following as independent variables: maternal race, marital status, maternal place of birth, maternal education level, maternal employment status, access to transportation, maternal diabetes, maternal high cholesterol, maternal body mass index, maternal perceived health status, maternal vegetable consumption, and food insecurity.

## Discussion

In this sample of low-income WIC recipients in a large urban area, characteristics independently associated with farmers market use or intention to use farmers markets were reporting maternal history of diabetes, maternal unemployment, food insecurity, and maternal vegetable consumption. These findings provide a unique snapshot of the factors associated with farmers market use in a diverse population of urban low-income families.

Although substantial work has established a relationship between neighborhood built environment and community obesity prevalence (3), no prior study has assessed the relationship between maternal health status and farmers market use among WIC recipients. Our findings challenge other ecologic studies that have linked community characteristics (eg, supermarket proximity, neighborhood walkability) with improved diet-related health outcomes (19). One study found that the density of farmers markets was inversely related to county-level obesity prevalence, even after adjusting for income level, race, and ethnicity (20). In light of this evidence, it might seem surprising that in our sample, WIC mothers who attended farmers markets were significantly more likely to have diabetes than those who did not attend. The literature has focused primarily on how factors like neighborhood farmers markets affect community-level health status. In contrast, our study looked at the individual-level factors associated with farmers market use within a diverse, low-income community. We hypothesize that mothers of young children who have diet-related illnesses may be most likely to seek increased fresh vegetables in their family’s diet. These mothers may be unique in perceiving exceptional benefits from increased access to farmers markets and other convenient sources of fresh produce.

Our findings that maternal unemployment, lower access to automobile transportation, and lower food security were associated with an expressed intention to use farmers markets use are at odds with previous studies. A study of low-income adults in the rural South, for example, found that respondents with a 2-year or 4-year college degree were significantly more likely to shop at farmers markets (10). An earlier study found that farmers market participants in California were older, middle-to-high income, married, and part-time employed or unemployed (21). A third study found that income level was not associated with the probability of shopping at a farmers market (9). One study found no relationship between participation in the WIC FMNP and food security status (22). A series of focus groups conducted with food stamp recipients in Oregon found that farmers markets were perceived as too expensive, especially when compared with traditional grocery stores (8).

The findings presented in this study about the vector of the relationship between socioeconomic status (SES) and farmers market participants have implications for public health professionals. Although our study examines a small convenience sample in 1 stressed urban environment, it provides important sentinel evidence that within low-income communities, farmers markets have a promising likelihood of reaching the most vulnerable populations. The finding that mothers who intended to shop at farmers markets were more likely to acknowledge financial barriers to feeding their children healthy foods is especially remarkable in an urban area such as Miami, Florida, where the poverty rate is 27.7% (compared with a national average of 14.3%) and the child poverty rate is 38.2% (compared with a national average of 20.0%) (11). Although a continued focus on providing affordable healthful options for low-income populations is imperative, farmers markets should not be discounted as too expensive a setting for implementing interventions aimed at improving diet-related health outcomes.

Our findings that first-generation immigrants and high-vegetable consumers were more likely to use a farmers market were consistent with prior public health observations. Hispanic people living in the United States with low levels of language acculturation are more likely to consume fruits and vegetables and have healthier overall dietary habits than their more acculturated counterparts (23,24). In an Ohio-based study, mothers in the WIC FMNP group reported significantly higher vegetable (but not fruit) consumption than mothers in the WIC-only group (22).

Our findings are subject to limitations common to small observational studies. Given the single setting and convenience-sampling design, our findings are not generalizable to all urban environments. For immigrant populations, the study results may be affected by selection bias, given that Latinas were undersampled relative to the clinic population. Reporting bias and social desirability bias may interfere with interpretation of self-reported health status and health behaviors. We were unable to report a numerical participation rate; however, we do not believe the lack of a participation rate compromises the validity of the study findings. Study procedures were explicitly designed to reduce the chance of nonparticipation bias and included use of broad eligibility criteria, approaching all women in the clinic who were 18 or older at time of study recruitment, administering the survey in English and Spanish, and offering a small monetary incentive to all participants. Reported SES of mothers in our study was comparable to reported SES in a study conducted with 290 caregiver/child dyads at the same urban public hospital. In that study, 24.4% of caregivers had not completed high school (25). Additionally, a review of participation rates in epidemiologic studies by Galea and Tracy noted that “most studies have found little evidence for substantial bias as a result of nonparticipation” (26). Although bias from nonparticipation may result when respondents and nonrespondents differ on variables of interest, studies on nonparticipation have found few significant differences between respondents and nonrespondents (26).

Despite these limitations, this study has numerous particular strengths. Interviewer administration of the survey instrument may have helped overcome some of the limitations of survey self-completion, reducing selection bias and increasing inclusion of low-literacy and limited-English-proficiency respondents. This study was the first of its kind to collect detailed data on family-health factors associated with farmers market use in a diverse, low-income population of WIC recipients. The clinic population from which participants were recruited was racially and ethnically diverse, like much of urban Miami-Dade County.

This study provides unique insight into the characteristics associated with farmers market use in a low-income population not easily reached by prior survey methods. The community-based nature of the study design was a strength. The study was developed as part of a community-wide partnership, involving public health practitioners, nutritionists, parents, farmers, health care providers, community–child health advocates, and WIC providers. These partners made contributions to every step of the research process, from designing the survey instrument to analyzing study results.

This study has numerous implications for policy makers. A new report from the Institute of Medicine showed that Americans aged less than 50 years face a significantly higher burden of diabetes and cardiovascular disease compared with young citizens of 16 other high-income countries (27). Innovative approaches are needed to address this growing public health challenge. Farmers market–based interventions may be an effective strategy to improve community wellness among low-income populations. A study of WIC recipients in Los Angeles found that farmers market participants increased their consumption of fruits and vegetables by 1.4 servings per 1,000 calories from baseline to the end of intervention compared with controls (28).

Our findings that farmers market participants were more likely to have diabetes, be food insecure, and be unemployed only magnify the potential impact of such interventions. We hypothesize that the populations that stand to benefit the most from increased access to fresh fruits and vegetables may be most susceptible to the benefits of the WIC FMNP. Given the need for novel strategies to reduce rates of diet-related chronic conditions, this study provides evidence that farmers markets can reach low-income families at high risk for poor diet-related health outcomes. Future studies should further explore the efficacy of farmers market-based interventions at improving dietary behaviors and related health outcomes in low-income populations.
